# Precise limb-speed afference reveals rhythmogenic drive modality and declines with age

**DOI:** 10.21203/rs.3.rs-7594824/v1

**Published:** 2025-09-17

**Authors:** Emily Herrick, Erienne Olesh, Cheryl Brandmeir, Sergiy Yakovenko

**Affiliations:** 1 Department of Biomedical Engineering, Benjamin M. Statler College of Engineering and Mineral Resources, West Virginia University, Morgantown, WV, USA; 2 Department of Human Performance – Exercise Physiology, School of Medicine, West Virginia University, Morgantown, WV, USA; 3 Department of Neuroscience, School of Medicine, West Virginia University, Morgantown, WV, USA; 4 Department of Human Performance – Physical Therapy, School of Medicine, West Virginia University, Morgantown, WV, USA; 5 Department of Mechanical and Aerospace Engineering, Benjamin M. Statler College of Engineering and Mineral Resources, West Virginia University, Morgantown, WV, USA; 6 Rockefeller Neuroscience Institute, School of Medicine, West Virginia University, Morgantown, WV, USA

## Abstract

Human locomotion requires precise coordination of bilateral limb movements that degrades with age increasing frailty and loss of independence. Here we tested whether the nervous system senses small differences in limb speed and whether this signal is independent of loading and changes across the lifespan. Using a two-alternative forced-choice task on a split-belt treadmill, healthy young adults detected inter-limb speed differences as small as 1.3% (mean 1.3%, standard deviation 0.6%). Manipulating whole-body load by ±10% did not alter thresholds, consistent with computation of whole-limb speed rather than raw somatosensory drive. Across ages 6–80 years, discrimination followed a U-shaped trajectory: thresholds were higher in children, lowest in young adults, and increased in middle-aged and older adults. Here, we show that limb-speed afference is measured with high acuity in humans and declines with age, supporting its role as a control variable for rhythmogenic circuits and highlighting a potential target for assessment and rehabilitation in age-related mobility decline.

## Introduction

Maintaining mobility is central to healthy aging, yet gait impairments are among the earliest and most disabling consequences of biological aging (Studenski, 2011). Slowed gait speed and reduced adaptability are strongly predictive of falls, frailty, hospitalization, dementia, and mortality, making locomotor performance one of the most powerful clinical biomarkers of aging (Abellan Van Kan et al., 2009; Pulignano et al., 2016). Despite its importance, the neural computations that support stable walking across the lifespan remain poorly understood (Wilson et al., 2019); real-world measures related to pace and mobility are needed in older adults (Albites-Sanabria et al., 2025).

Efficient bipedal locomotion requires the nervous system to generate rhythmic patterns of muscle activity while flexibly adapting to environmental demands. A long-standing framework posits a hierarchical architecture in which supraspinal centers set global goals (e.g. walking speed) and segmental spinal networks—central pattern generators (CPGs) together with sensory feedback—shape the coordination of muscle activations (Grillner, 1985; Kiehn, 2016; Prochazka and Yakovenko, 2007; Rybak et al., 2024). The simple control law links the different computational reference frames: *desired limb-endpoint speed is the primary input that entrains CPG rhythm* (Danner et al., 2017; Yakovenko et al., 2005). The supporting evidence comes from the experimental studies that reveal that even a single channel of ramp stimulation in midbrain nuclei can control high-dimensional coordinated locomotor activity from stepping to galloping, suggesting the speed control (Shik et al., 1966).

The computational studies further reveal mechanistic transformation through the CPG. By solving inversely—from the outputs to inputs—a relatively simple CPG model expressed as the system of nonhomogeneous state equations and constrained by experimental data, we identified that limb speed is linearly related to the CPG input (Yakovenko, 2011). Thus, limb speed is the *driving* control integrated within a flow of visuomotor transformations to control heading direction (Yakovenko et al., 2018). The link between heading direction (γ) and limb speed difference (γ) can be described by a kinematic circular path with approximated step geometry defined by the step cycle duration ($dV\;=\;V_{R}-V_{L}$) generated by the CPG, and the effective step width $(W):\;\gamma \;\approx \frac{T_{c}(V_{R}-V_{L})}{W}\;$ (Courtine et al., 2006; Patla et al., 1999). Inverting this relationship, two possibilities exist for steering by the supraspinal controller: (i) using limb speeds to drive the CPG, or (ii) sending a common drive with transient asymmetric biasing of the rhythmogenic circuitry by the interlimb speed difference (dV). The two mechanisms are functionally equivalent, yet the evidence for this control in humans has not been previously described.

The idea that the neural computations command a dynamic mechanical system with the purpose of maximizing certain performance variables aligns with the optimal feedback control hypothesis (Scott, 2004; Todorov and Jordan, 2002). According to this view (see Figure 1), the descending inputs from supraspinal centers are tuned by continuous sensory feedback to minimize biologically relevant cost. The detected errors between desired and observed signals are continuously minimized to maintain accurate predictive and reactive control of locomotion (Darici and Kuo, 2022; Kuo, 2002). The classic control theory offers an additional helpful insight; it suggests that the state variables must also be observable for optimal regulation and tracking (Kalman, 1960; Ogata, 2020). Thus, the hypothesis that limb speed is the *efference* that *drives* the spinal rhythmogenic networks can be restated as the expectation of precise limb-speed *afference*, the sensory feedback of efferent signal, in humans. The acuity of the specific sensory feedback available at the high levels of neural hierarchy can be consciously observed and tested.

The mechanical state of the body is reported through proprioception, kinesthesia, and sensory feedback, which are not fully interchangeable and terms (Prochazka et al., 2000; Proske and Gandevia, 2012). Proprioception and kinesthesia require *conscious* sensation of movement, while sensory feedback is a general mechanistic term that also includes unconscious (automatic) signals. The conflated view on proprioception and kinesthesia defines this human ability to observe sensory feedback at low and high hierarchical levels of processing error-correction. Prochazka (Prochazka et al., 2000) suggested that proprioception is “providing us with a glimpse of internal shifts of conscious attention in the brain as movements shift back and forth along the *reflex-voluntary* continuum.” Thus, psychophysical methodology can be used to examine this operation across different functions and structures. Surprisingly, the evidence for whole-limb or body-acuity is scarce; prior work has primarily focused on sensory modalities of proprioception, such as joint position and force (Allen et al., 2007; Gritsenko et al., 2007). However, integrated kinesthetic signals, such as subject speed perception (Lalonde-Parsi and Lamontagne, 2015), vertical orientation (Bisdorff et al., 1996; Fraser et al., 2015), and limb end-point position (Rincon-Gonzalez et al., 2011; Van Beers et al., 1998) are less established and rarely explained within a mechanistic theoretical framework. If the limb speed is a control parameter, then it is likely that humans can demonstrate high acuity to limb-speed afference, which is the focus of this study.

Another important consideration is the examination of the limb-speed acuity across the lifespan. During childhood, sensory-motor maps are refined through the increased reliability of sensory responses (Holst-Wolf et al., 2016), whereas aging degrades proprioceptive precision and locomotor adaptability (Osoba et al., 2019). We therefore hypothesized that limb speed afference would follow a parabolic trend, peaking in young adulthood. While the presence of this theoretical relationship is observed across many sensorimotor variables, such as reaction time (Marusic et al., 2022; Toledo et al., 2016), sensory acuity (Goble et al., 2009; Skedung et al., 2018), and even overall happiness (Galambos et al., 2020), the specific profile of limb-speed acuity has not been demonstrated.

Here, we test three hypotheses that investigate the processing within the locomotor control networks. First, humans have high acuity for interlimb speed as detected by the proprioceptive threshold. Second, this threshold does not depend directly on the sensory output, as demonstrated by whole-body loading and unloading, indicating the computation of a high-level parameter. Third, the limb-speed acuity changes from childhood to older adulthood. Our results provide the empirical support for finely tuned limb‑speed afference and its lifespan dynamics, substantiating the role of this signal as a control variable in human gait. The partial preliminary results were published in abstract form (Galbreath et al., 2014).

## Results

### Detection and Discrimination of Limb Speed Differences.

We tested the general idea that humans use limb speed as the efferent input signal to drive spinal rhythmogenic circuits. While the direct testing of this theoretical concept is limited in intact locomotor system, we used a consequent hypothesis that the precise control signal is also reflected in the precise afference of this signal. Thus, we tested whether young adults could sense the precise limb speed while walking on a split-belt treadmill. Our analysis of outcomes in a forced binary psychophysical task revealed the thresholds of limb speed afference.

**Figure 2.**
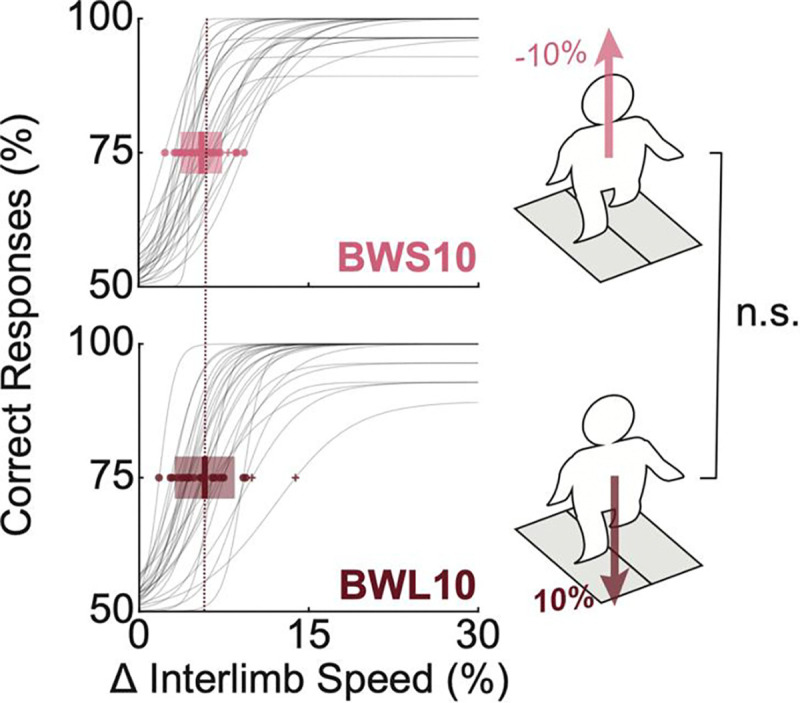
Comparison of JND_75_ across loading conditions. Psychophysical curves illustrating the percentage of correct responses versus interlimb speed difference (dV) for individual participants under −10% and +10% loading conditions (BWS10, BWL10). Gray curves show individual performance, with highlighted points indicating individual JND_75_ values. Bold lines and shaded areas represent group means ±1 SD. The dashed red reference line indicates the mean JND_75_ for the BWL10 loading condition, facilitating visual comparison across conditions. Outliers (Tukey’s 1.5×IQR) are indicated (+).

Figure 2 illustrates the psychophysical task and measurements of the estimated detection threshold. The individual logistic curves were fitted into the relationship between the percentage of correct responses and interlimb speed differences for participants in the young adult (YA) group. The success rate values of correct responses were fitted with a sigmoidal logistic curve to identify the slope inflection points, which typically correspond to the steepest slope at 75% success rate or just noticeable difference at 75% (JND_75_). However, in a two-alternative forced-choice task, the performance by chance is 50% correct responses (the floor of the of the logistic curve), which prevents the empirical measure of a detection threshold. As such, Figure 2B illustrates the tangent line approximation at the highest slope, to linearly extrapolate the function back to an estimated point where the success rate would drop to 50% (extrapolated or E-JND_50_). Figure 2C shows the distribution of the JND_75_ values across the young adult cohort. Figure 2D&E show the distribution of E-JND_50_ values across the young adult cohort, and the data was normally distributed (Kolmogorov-Smirnov test, p = 0.99). The mean E-JND_50_ for limb speed was **1.29 ± 0.60%** (mean ± SD).

### Influence of Loading Conditions.

Figure 3 shows psychophysical curves and distributions of JND_75_ across 10% body-weight support (BWS10) and 10% body-weight loaded (BWL10) conditions. Data across all conditions met normality criteria (Kolmogorov-Smirnov test, BWS10: p = 0.94, BWL10: p = 0.76). Mean JND_75_ values (± SD) were as follows: 5.43 ± 1.85% for BWS10 and 5.31 ± 1.94% for BWL10 conditions. An equivalence test comparing the BWS10 and BWL10 conditions revealed a small effect size (0.06), below the predefined equivalence margin of 0.2, confirming equivalence between these two conditions.

### Age-Related Changes in Limb Speed Discrimination.

**Figure 4.**
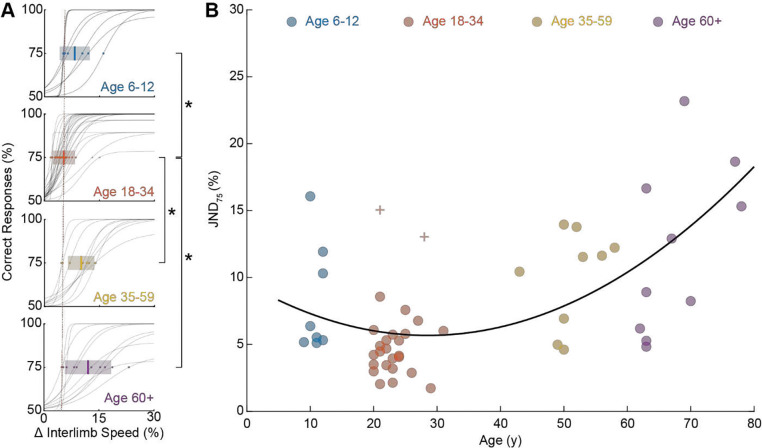
Differences in limb speed discrimination (JND_75_) across the human lifespan. **A.** Psychophysical curves showing percentage correct versus interlimb speed difference across age groups: children (**blue**), young adults (**red**), middle-aged adults (**yellow**), and older adults (**purple**). Individual participant data are shown as gray curves, with highlighted JND_75_ values. Bold lines and shaded areas indicate group means ±1 SD. The dashed red reference line denotes the young adult group mean, highlighting age-related shifts. Outliers (Tukey’s 1.5×IQR) are marked (+), and significant differences are indicated (*, p < 0.05). **B.** Scatter plot illustrating JND_75_ versus age, fitted with a parabolic regression line.

Figure 4A shows individual psychophysical curves and distributions of JND_75_ across different age groups. Normality conditions were satisfied for all groups (Kolmogorov-Smirnov test; children: p = 0.39, young adults: p = 0.99, middle-aged adults: p = 0.70, older adults: p = 0.80). Mean JND_75_ values (± SD) were 8.23 ± 4.10% for children, 4.56 ± 1.74% for young adults, 10.01 ± 3.60% for middle-aged adults, and 12.02 ± 6.30% for older adults. A one-way ANOVA identified significant differences among age groups (p < 0.01). Post-hoc analyses (unpaired, left-tailed t-tests with Holm-Bonferroni correction; the left-tailed test type was justified by the pre-specified hypothesis of reduced performance with age) confirmed significant differences between young adults and all other groups (children: p < 0.01; middle-aged adults: p < 0.01; older adults: p < 0.01), but not between middle-aged and older adults (p = 0.21). Figure 4B illustrates the relationship between JND_75_ and age, described by a parabolic fit (equation: $y=4.76\cdot 10^{-5}x^{2}-0.003x+0.10$).

## Discussion

Our findings demonstrate that humans perceive inter-limb speed differences with remarkable precision, and this capacity changes across the lifespan. The U-shaped trajectory, with peak acuity in young adulthood and deterioration in older age, parallels lifespan profiles observed in other sensorimotor and cognitive domains. Importantly, limb-speed acuity was unaffected by ±10% load manipulations, underscoring that it represents a high-level neural computation rather than low-level afferent inflow. This robustness makes limb-speed perception an attractive candidate for clinical translation.

Age-related increases in thresholds may reflect multiple mechanisms: reduced fidelity of spinocerebellar pathways, loss of muscle spindle density, cognitive problems, or integrative deficits in visuomotor transformation. Regardless of mechanism, the decline of this signal aligns with the broader phenotype of age-related mobility loss, frailty, and fall risk. Thus, limb-speed acuity could provide a mechanistic bridge linking neural control theories of locomotion with clinical biomarkers of aging. We drew theoretical predictions from the previous computational and experimental studies predicting limb-speed efference to control walking with turning (Courtine and Schieppati, 2003; Patla et al., 1999; Yakovenko, 2011; Yakovenko et al., 2018) and were inspired by the classical control theory idea that a controlled variable must be accurately observed to form a consequent hypothesis that humans have an accurate sense of limb speed during walking. Our results indicate that humans can report minute changes in limb speeds, as evidenced by the low threshold of 1.29% in interlimb speed differences; moreover, the threshold value remains insensitive to the manipulation of proprioceptive drive through whole-body loading and unloading. This independence of limb-speed afference is consistent with its high-level hierarchical representation within the locomotor control pathways. Additionally, the acuity to limb-speed afference throughout the human lifespan follows a quadratic U-shaped relation with age, tracking the fine-tuning of this function during development and its deterioration in the aging stages of life.

The central idea underlying the hypotheses of this study is that efferent commands may contain different signal modalities, such as a high-level representation of limb speed, alongside detailed commands to modify the activity of specific muscle groups. Historically, high-level commands were thought to be represented within neural pathways. Descartes likened the control of limb motion to the hydraulic operation of water in fountains. He imagined a general driving source manipulated through switches to produce the desired output. The general match between neural processing and the fountain analogy is admittedly influenced by post-hoc knowledge, as the concepts of rate coding were unknown at the time, and the literal imagery of fluid dynamics operations is fundamentally different from the neural integration of spatiotemporal activity patterns. The concept of dynamic neural computations with rate models has been valuable for a wide range of sensorimotor processes and mechanisms, including motor learning and planning (Shadmehr et al., 2016; Wolpert et al., 2011), and normal and damaged locomotor pattern generation (Pryyma and Yakovenko, 2022; Verzár, 1923). In particular, Verzár used a pair of linked vessels to simulate hydraulically the operation of Brown’s conceptual half-center oscillator model of CPG (Brown, 1911), which is, to our knowledge, the first demonstration of neural simulation with experimentally observed behavior.

The evidence of both high-level and low-level control is evident in the discharge of neural populations of primary motor cortex neurons, shown to encode high-level kinematic variables such as end-point limb speed (Moran and Schwartz, 1999) as well as precise muscle group control in reaching, precise stepping, and obstacle avoidance tasks (Drew et al., 2008; Holdefer and Miller, 2002; Overduin et al., 2012). The sophistication of sensory responses also increases with the latency of processing and captures the whole limb’s mechanical state in response to perturbations (Omrani et al., 2014; Pruszynski et al., 2011). The low-level computations, including closed-loop interactions with sensory inflow, may use muscle force and/or equilibrium length as the computational reference frames. The event-driven and continuous examples of this are, respectively, the triggering of perturbation-driven change in muscle recruitment (Dietz and Duysens, 2000) and continuous contributions to the ongoing locomotor activity (Yang et al., 1991). The nonlinear comprehensive details of the primary afferent drive to individual muscles during walking can be simulated using neuromechanical models (Yakovenko et al., 2004). These signals converge on the CPG to prolong or curtail the corresponding locomotor phases (Rossignol et al., 2006) or contribute to the descending locomotor drive, which changes locomotor velocity (Yakovenko et al., 2004). Overall, the sensory system enables high-level decisions to influence the ongoing activity, for example, by increasing the efferent drive (Yakovenko, 2011) or, alternatively, by increasing the ensemble gain of sensory feedback—afference—speeding up locomotion (Prochazka et al., 2002). Thus, the closed-loop sensory feedback can activate single motoneuron pools and also express the whole-limb commands in the reference frame of inputs to the CPG. Both efferent and afferent signal modalities reflect a hierarchical organization and the capacity to encode multiple agglomerations of actions.

Since the sensorimotor control of movement is often described as an optimal control system (Scott, 2004; Todorov and Jordan, 2002), there should exist a relationship between efferent and afferent signal modalities. The consequence of expecting a high-level efferent control signal is the hypothesis that the high-level afferent signal is independent of the low-level sensory inflow. We predicted that the proprioception of limb-speed threshold would be independent of changes in low-level sensory feedback due to whole-body loading or unloading. Thus, the changes in loading are expected to alter cutaneous, primary, and secondary afferent discharge, thereby modifying the EMG activity of extensors during standing and walking (Dietz and Colombo, 1998), but do they modulate the high-level proprioception?

The results in Figure 3 support this hypothesis, showing that neither whole-body loading nor unloading changed the limb-speed perception threshold. Evidence for a similar high-level sensory representation has been reported within the rapid afferent spinocerebellar pathways (Bosco and Poppele, 2000). The sensory processing related to the limb end-point state was shown in Clark’s column neurons within a discrete thoracic-lumbar nucleus (Edgley and Gallimore, 1988) that gives rise to the dorsal spinocerebellar tract, which rapidly delivers this sensory modality to the cerebellum. Clarke’s column lies in Rexed’s lamina VII of the lumbar and thoracic spinal cord segments and integrates heterogeneous spindle and, to a lesser extent, Golgi tendon and secondary afferent projections (Bosco and Poppele, 2001). Moreover, both dorsal and ventral spinocerebellar tract neurons were shown to receive the CPG corollary discharge (Fedirchuk et al., 2013; Stecina et al., 2013). While the exact function of this circuit within Clark’s column remains unclear, the fact that the efferent copy of the CPG drive is available within this circuit indicates its high capability for estimating limb speed based on the desired and observed signal modalities. Additionally, a recent study demonstrated that young adults can perceive subtle slip-like disturbances to their walking balance even when they are cognitively distracted (Liss et al., 2022). Thus, our results are consistent with the idea that humans use limb speed within the locomotor control system.

Our original computational analysis, which formed the hypotheses of this study, examined the neural computations involved in the visuomotor transformation for spatial navigation, specifically from the heading direction to the CPG modulation of ongoing locomotor phases (Yakovenko et al., 2018). For coordinated movements in cluttered environments, vision provides information on heading direction, size, shape, and distance to objects processed to generate and execute timely motor plans, for example, to step over obstacles. The neural dynamics of the spinal CPG are modified for guided locomotion like turning, speed change, or stepping over obstacles. These types of descending control interventions require processing in the premotor and motor cortices (Lajoie et al., 2010; Lajoie and Drew, 2007). The “easy task” of stepping over an obstacle requires engaging a top-down hierarchy to process visual information (as reviewed in Drew et al., 2008) and integrate environmental external constraints and demands, as well as neuromechanical internal dynamics defined by the bottom-up organization (Yakovenko, 2011). The predictive part of this transformation has been previously described in terms of the canonical mechanism of grid and speed cells. In rodents, medial entorhinal grid cells encode heading direction and velocity tuning, providing a conjunctive state estimate for path integration (Sargolini et al., 2006). A distinct population of speed cells supplies a linear, prospective code for velocity magnitude that feeds back into the grid network, keeping the temporal synchronization and scaling of the heading vector (Kropff et al., 2015). During planning, posterior parietal neurons integrate allocentric heading information with egocentric visual targets and limb mechanical states, performing reference-frame transformations that predict upcoming movement trajectories (Buneo et al., 2002; Buneo and Andersen, 2006). These findings outline a hierarchical circuit in which grid and speed cells supply a dynamically updated limb-velocity vector that parietal visuomotor areas recast into effector-specific commands, thereby supporting the view that limb-speed signals are the output to the execution pathways.

The evidence of precise tuning of the limb-speed processing within the locomotor control system indicates the central role of this modality in human motor development and age-related deterioration. Consequently, we predicted a parabolic relationship between limb-speed threshold and age, which was supported by our observation in Figure 4. During childhood and adolescence, speed-related sensorimotor circuits are progressively calibrated. Proprioceptive position-sense acuity improves until mid-adolescence, reaching adult‑like levels at 14 years old (Holst-Wolf et al., 2016). Additionally, stride-to-stride variability in gait velocity declines steadily from preschool age to puberty, reflecting tighter neural control over locomotor speed (Hausdorff et al., 1999). Conversely, later in life, age-related mobility dysfunctions encompass a range of impairments in gait, balance, and motor control, which can severely impact the quality of life and independence of older adults. These issues stem from neuromuscular and sensory declines that weaken overall mobility and coordination. Age-related sarcopenia, or muscle loss, reduces functional capacity and heightens fall risk (Cruz-Jentoft et al., 2010). Structural changes in tendons lower stiffness, which destabilizes joints and alters gait mechanics (Narici and Maffulli, 2010). Meanwhile, sensory signaling from the body’s proprioceptors, such as afferents originating in muscle spindles and tendon organs, declines, further disrupting balance and coordination (Goble et al., 2009). Combined, these changes increase the metabolic cost of walking, as older adults must recruit alternative muscles, specifically those around the hip joint, to maintain stability (Ko et al., 2011) thus reducing mobility with age (Mian et al., 2006). This higher energy demand is associated with decreased endurance and a lower likelihood of sustained physical activity, which accelerates physical decline and heightens injury risks (Lauretani et al., 2003). This general sensorimotor decline may be exacerbated by disruptions in high-level signal processing within the spatial navigation system. Overall, our results are consistent with the lifespan changes in limb-speed representation within the locomotor system.

In summary, limb-speed perception is a fundamental neural control signal for locomotion that is robust to sensory manipulations yet declines with age in a characteristic U-shaped trajectory. These findings position limb-speed acuity as both a mechanistic insight into locomotor control and a practical biomarker for age-related decline in mobility. Incorporating limb-speed testing into clinical and rehabilitation settings may enable earlier detection of mobility impairments and inform interventions aimed at preserving independence across the aging lifespan.

## Methods

### Participants.

Healthy participants (N=53) were recruited into four age groups: children (6–12 yrs; N=8, mean 10.9 ± 1.1 yrs; 7 females), young adults (18–34 yrs; N=26, mean 23.3 ± 2.9 yrs; 12 females), middle-aged adults (35–59 yrs; N=9, mean 51.2 ± 4.3 yrs; 5 females), and older adults (≥60 yrs; N=10, mean 67.5 ± 6.0 yrs; 3 females). All procedures were approved by the Institutional Review Board of West Virginia University, and informed consent was obtained from all adult participants, and parental consent was obtained for child participants.

### Experimental Paradigm.

Participants walked on a split-belt treadmill designed to impose randomized interlimb speed differences (0%, 2%, 5%, 12.5%, 20%, and 30% relative to participant-specific preferred walking speeds; see Figure 2A). Participants, wearing glasses to obscure ground view, were asked to determine which limb moved faster based solely on proprioceptive feedback, with vision occluded using visual-field-limiting glasses. Each trial consisted of a 3-second proprioceptive evaluation phase initiated by an audio cue (“Focus”), followed by a verbal response indicating the faster limb (“Report”). Each speed condition was repeated seven times, with rest breaks provided as requested due to task duration (~20 min). Young adult participants additionally completed two loading conditions: 10% body-weight support (BWS10) and 10% body-weight loading via backpack weights (BWL10).

### Detection Threshold Analysis.

Only young adult participants were included to establish baseline limb-speed detection thresholds. For each participant, response accuracy was computed at each interlimb speed difference and converted to percentage-correct values. Probabilities ≤50% were set to 50%, reflecting the chance level due to the binary choice. The probability values were normalized to [0,100] and fitted with logistic functions ($P_{\text{fit}}$):

(1) $$P_{fit}(x)=1/\left(1+e^{-k\left(x-x_{0}\right)}\right)$$

where x is the interlimb speed difference (%), k the slope, and $x_{0}$ the inflection point.

Detection thresholds were defined as the intersection of the tangent line at the inflection point with the x-axis. Negative thresholds were excluded as physiologically meaningless. Outliers beyond Tukey’s 1.5×IQR criterion were removed. Normality was tested using Kolmogorov–Smirnov tests.

### Influence of Loading Conditions.

Young adult data from BWS10 and BWL10 conditions were compared to determine whether limb speed sensing depends directly on limb loading. JND_75_ values were determined from logistic fits. Tukey’s 1.5×IQR method identified outliers. Normality assumptions were verified (Kolmogorov–Smirnov test). Equivalence testing between BWS10 and BWL10 conditions was conducted, with equivalence defined as Cohen’s d<0.2.

### Age-Related Changes in Limb Speed Discrimination.

Age-related differences in limb speed perception were examined across all groups using JND_75_ values. Participants unable to achieve 75% accuracy were excluded. Normality assumptions were verified. Mean JND_75_ values were compared using a one-way ANOVA followed by Holm–Bonferroni-corrected post-hoc left-tailed t-tests. Additionally, to quantify continuous age effects, quadratic regression analyses were performed between age and JND_75_.

## Figures and Tables

**Figure 1.**
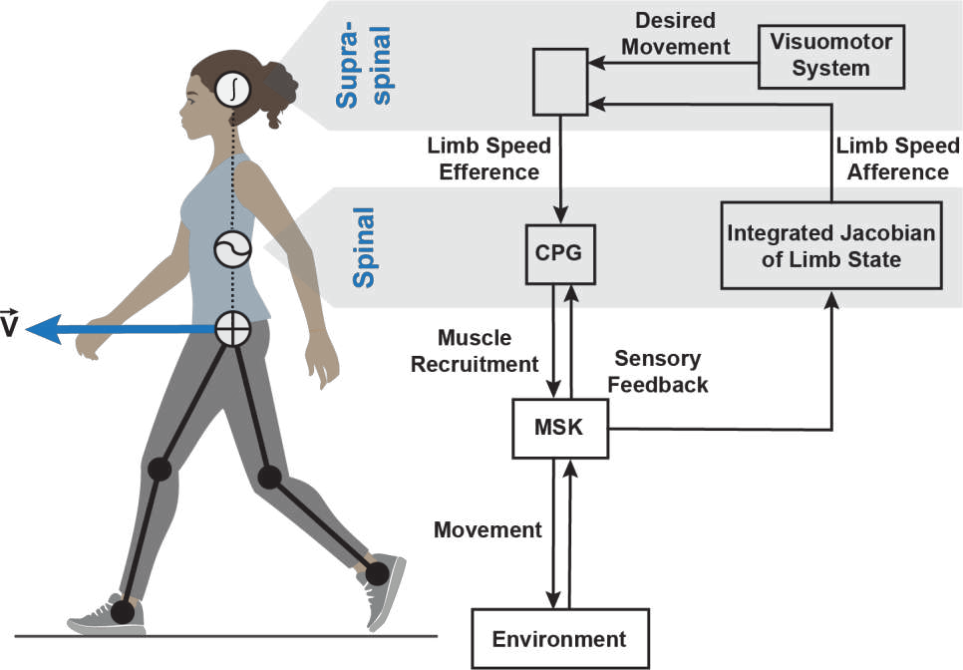
Conceptual framework for limb-speed signaling within the hierarchical sensorimotor system. Visuomotor circuits provide commands such as heading direction to compute limb-speed efferent signal driving rhythmogenic spinal circuits, the central pattern generator (CPG). The output is muscle recruitment signals processed through the musculoskeletal (MSK) transformation, generating body movement. Concurrent sensory feedback from cutaneous and proprioceptive afferents is integrated to represent limb state at the supraspinal levels as limb-speed afference.

**Figure 1.**
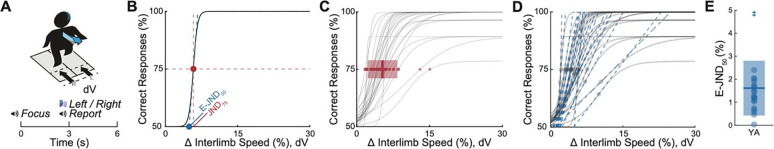
Limb speed detection thresholds in young adults. **A.** Psychophysical testing of limb speed perception in a forced binary task. Subjects experience a difference in belt speeds for 3s and are asked to report, which leg was faster—left or right—within the next 3s. **B.** Extrapolated just-noticeable-difference (E-JND) is derived from the slope of the logistic curve and its intersection with the line of 50% correct responses. Estimated thresholds E-JND_50_ are lower than JND_75_ values. **C**. Individual psychophysical curves depicting the percentage of correct responses as a function of interlimb speed differences, with the distribution of JND_75_ values superimposed. Highlighted points indicate individual JND_75_ values. The bold line and shaded area represent the group mean ±1 SD. **D.** Individual psychophysical curves depicting the percentage of correct responses as a function of interlimb speed differences, with inflection points (gray points) and slopes at those points (blue dashed lines) superimposed. **E.** The individual detection thresholds are shown by the distribution of E-JND_50_ values (blue points), the bold line and shaded area indicate the group mean ±1 SD. Outliers identified using Tukey’s 1.5×IQR criterion are marked (+). Young adult (YA) group included individuals 18–34 yo.

## Data Availability

All source data underlying the figures and statistical analyses will be available in a Figshare repository upon publication.
